# High-Dimensional Mediation Analysis With Confounders in Survival Models

**DOI:** 10.3389/fgene.2021.688871

**Published:** 2021-06-28

**Authors:** Zhangsheng Yu, Yidan Cui, Ting Wei, Yanran Ma, Chengwen Luo

**Affiliations:** ^1^Department of Bioinformatics and Biostatistics, School of Life Sciences and Biotechnology, Shanghai Jiao Tong University, Shanghai, China; ^2^SJTU-Yale Joint Center for Biostatistics, Shanghai Jiao Tong University, Shanghai, China; ^3^Clinical Research Institute, Shanghai Jiao Tong University School of Medicine, Shanghai, China

**Keywords:** high-dimensional mediators, confounders, survival model, mediation analysis, propensity score

## Abstract

Mediation analysis is a common statistical method for investigating the mechanism of environmental exposures on health outcomes. Previous studies have extended mediation models with a single mediator to high-dimensional mediators selection. It is often assumed that there are no confounders that influence the relations among the exposure, mediator, and outcome. This is not realistic for the observational studies. To accommodate the potential confounders, we propose a concise and efficient high-dimensional mediation analysis procedure using the propensity score for adjustment. Results from simulation studies demonstrate the proposed procedure has good performance in mediator selection and effect estimation compared with methods that ignore all confounders. Of note, as the sample size increases, the performance of variable selection and mediation effect estimation is as well as the results shown in the method which include all confounders as covariates in the mediation model. By applying this procedure to a TCGA lung cancer data set, we find that lung cancer patients who had serious smoking history have increased the risk of death *via* the methylation markers cg21926276 and cg20707991 with significant hazard ratios of 1.2093 (95% CI: 1.2019–1.2167) and 1.1388 (95% CI: 1.1339–1.1438), respectively.

## Introduction

Mediation analysis was firstly used to deal with the causal chain of events as the primary exposure has an effect on the outcome through affecting one or more mediators in psychological studies, and gradually extended to sociological and biomedical researches (Baron and Kenny, 1986; MacKinnon et al., 2002; Preacher and Hayes, 2008; Biesanz et al., 2010; Huan et al., 2016). Of note, the mediators are usually measured after the intervention, but before the main outcome of interest. Mediation effect is often assessed through a regression-based analysis procedure by decomposing the total effect that describes the relationship between the exposure and the outcome variable into direct effect and indirect effect (Baron and Kenny, 1986; MacKinnon et al., 2007). In the past couple of decades, the topic of mediation analysis has received a great deal of attention, particularly in the area of causal inference (Robins and Greenland, 1992; Ten Have et al., 2007; Albert, 2008; Sobel, 2008; VanderWeele, 2009; Pearl, 2014). Researches in mediation analysis have been generalized from the case of a single mediator to multiple mediators (Albert and Nelson, 2011; Zhang and Wang, 2013; VanderWeele and Vansteelandt, 2014; Daniel et al., 2015), even to the case of high-dimensional mediators (Huang and Pan, 2016; Zhang et al., 2016; Zhao and Luo, 2016; Chén et al., 2018; Sohn and Li, 2019; van Kesteren and Oberski, 2019; Zhao et al., 2020). Recently, much progress has been made in extensive of mediation methods to survival models (Lange and Hansen, 2011; VanderWeele, 2011; Wang and Zhang, 2011; Huang and Yang, 2017).

The regression-based or structural equation modeling approach is commonly used to assess mediation effect. This approach assumes that there are no confounders influencing the relationships among exposure and mediator, mediator and outcome, and exposure and outcome. Randomization to levels of the exposure guarantees that there are no confounders that influence both the relation of exposure-mediator and exposure-outcome. However, the assumption that individuals are randomly assigned to exposure, especially for research about smoking and lung cancer, is difficult to achieve.

Propensity score method can be used to solve such a problem with a non-randomized exposure which usually appears in observational studies (Rosenbaum and Rubin, 1983). Previous studies have focused on mediation analysis with confounders in the case of a single mediator. For example, Valente et al. (2017) introduced confounders in mediation analysis and described how to address confounders with design-based techniques and analysis-based approaches. Coffman (2011) proposed to use the calculated propensity score to adjust for confounders between the mediator and the outcomes. However, methods for high-dimensional mediation selection adjusting for confounders, especially for survival outcome, are still yet to be developed.

For example, in a lung cancer study, it is showed that smoking increases the risk of lung cancer patients’ progression to death through DNA methylation markers (Luo et al., 2020). However, as an observational (or non-randomized) study, it is unrealistic for a subject to be randomly assigned to the exposure, as moral and ethical factors, in the research of how smoking affects the lung cancer patients’ risk of progression to death mediated by DNA methylations. Therefore, the relationship among smoking status, DNA methylations, and overall survival may be confounded by baseline characteristics, such as age, gender, and other physical health indicators. However, high-dimensional mediation analysis for survival analysis subject to confounders is still to be developed.

In this paper, we study mediator selection and indirect effect estimation *via* high-dimensional mediation analysis in survival models with confounders. For observational studies, as the exposure is not randomly assigned, we propose to use the propensity score approach to adjust confounding effects. The key ideas are as follows. Firstly, we adjust for baseline confounders based on the calculated propensity score which serves as a covariate in the mediation models. Secondly, we reduce the dimension of potential mediators from ultra high-dimensional to moderate (i.e., one that is less than the sample size) using sure independence screening (SIS) method (Fan and Lv, 2008). Thirdly, we conduct variable selection *via* Cox proportional hazards model with the minimax concave penalty (MCP) (Zhang, 2010). Finally, we carry out the Sobel and joint significance test for mediation effect.

The rest of the paper proceeds as follows. In the next part, we introduce the notations and models, definition of propensity score, and develop the proposed procedure. Then, we provide simulation studies to evaluate the performance of our proposed procedure and a real data application to analyze the mediation effects of high-dimensional DNA methylation markers on the causal effect of smoking on lung cancer in an epigenome-wide study. Finally, we conclude the paper through discussing limitations and other feasibilities.

## Statistical Method

### Notations and Models

**FIGURE 1:**
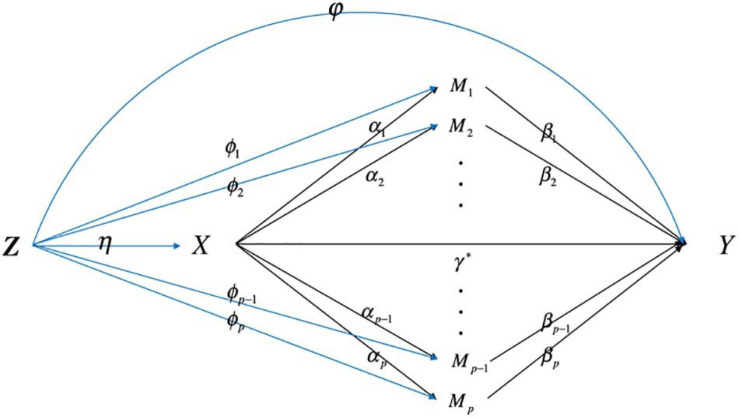
The directed acyclic graph describes high-dimensional mediation with confounders affecting the relation among exposure, mediator, and outcome.

For individual *i*,*i* = 1, 2,⋯,*n*, we let *D_i* denote the time from onset to an event (death) and *C_i* be the potential censoring time. The observed survival time is *T*_*i*_ = min(*D*_*i*_,*C*_*i*_), and the failure indicator is *δ*_*i*_ = *I*(*D*_*i*_≤*C*_*i*_), where *I*(⋅) is an indicator function. Let *X_i* be the exposure (smoking status, i.e., smoker or non-smoker), *M*_*i*_ = (*M*_1*i*_,*M*_2*i*_,⋯,*M*_*pi*_)^*T*^ be a p-dimensional continuous mediator vector (including all the methylation information), *p*≫*n*. In observational studies, the assumption that no confounders influence the relation of exposure-mediator, mediator-outcome, and exposure-outcome is violated. Let *Z* = (*Z*_1_,⋯,*Z*_*m*_)^*T*^ denotes for the baseline confounders. Figure 1 illustrates how confounders *Z* influence the relation of *X-M*, *M-Y*, and *X-Y*.

For survival outcome (Cox, 1972), the high-dimensional mediation models with confounders can be expressed as follows,

(1) $$\lambda_{i}\,\left(t\right)=\lambda_{0}\,\left(t\right)\,\exp\left\{\gamma^{*}\,X_{i}+\beta^{T}\,M_{i}+\varphi^{T}\,Z_{i}\right\},$$

(2) $$M_{k\,i}=c_{k}+\alpha_{k}\,X_{i}+\phi_{k}^{T}\,Z_{i}+e_{k\,i},k=1,2,\cdots ,p,$$

where Eq. (1) is the Cox proportional hazards model which describes the relationship between the exposure *X*, mediators *M* and the time-to-event variable; Eq. (2) characterizes how the exposure variables influence the mediators; *λ*_0_(*t*) is the baseline hazard function; *γ*^∗^ is the direct effect of the exposure on the outcome; *β* = (*β*_1_,⋯,*β*_*p*_)^*T*^ is the coefficient vector relating the mediators to the outcome adjusting for the effect of exposure and confounders; *φ* = (*φ*_1_,⋯,*φ*_*m*_)^*T*^ is the coefficient vector relating the confounders to the outcome; *α* = (*α*_1_,⋯,*α*_*p*_)^*T*^ is the coefficient vector relating the exposure to the mediators; *ϕ*_*k*_ = (*ϕ*_*k*1_,⋯,*ϕ*_*km*_)^*T*^ is the coefficient vector relating the confounders to the mediator; *c_k* is the intercept term; *e*_*ki*_∼*N*(0,*σ*^2^) is the residual.

### Propensity Score

The propensity score is proposed to help remove the selection bias result from potential confounders of *X* (Rosenbaum and Rubin, 1982). The propensity score is defined as the probability that an individual *i*, *i* = 1,⋯,*n* be allocated to the treatment group often estimated using logistic regression models, *π*_*i*_ = *Pr*(*X*_*i*_ = 1|*Z*_1*i*_,⋯,*Z*_*mi*_), given measured confounders *Z* = (*Z*_1_,⋯,*Z*_*m*_)^*T*^. This method is often used to minimize the influence of observed baseline covariates on the exposure. There are many propensity-based techniques for estimating average causal effect, including sub-classification (Rosenbaum and Rubin, 1984), matching (Rosenbaum and Rubin, 1985), and inverse propensity weighting (Robins et al., 1995). In this article, we focus on incorporating the calculated propensity score as the covariate to adjusting the confounding effects.

According to Rosenbaum and Rubin (1984), the propensity score is assessed by using baseline measured confounders as covariates in a logistic regression model with treatment status as the outcome as following

$$\text{logit}\left(P\left(X_{i}=1\right)\right)=\theta_{0}+\theta_{1}Z_{1\,i}+\cdots +\theta_{m}Z_{m\,i},$$

where *θ* = (*θ*_1_,⋯,*θ*_*m*_)^*T*^ denotes the coefficients of confounders on the exposure, and *θ*_0_ denotes the intercept. Hence, the propensity score, *π*_*i*_, the probability to be assigned to the intervention group can be expressed as

$$\pi_{i}=\frac{1}{1+\text{exp}\,\left(-\left(\theta_{0}+\theta_{1}\,Z_{1\,i}+\cdots +\theta_{m}\,Z_{m\,i}\right)\right)}.$$

The superiorities of propensity score over the classical regression adjustment method have been described elsewhere for the non-mediation model (Schafer and Joseph, 2008; VanderWeele, 2010). Briefly, propensity score approaches allow the inclusion of a large scale of confounders through reducing the potential covariates into a single numerical summary. More importantly, the comparison between subjects in treatment group and control group who have the same propensity score equals the comparison of control conditions with randomly assigned (Rosenbaum and Rubin, 1982).

### Methodology

Since the assumption of no confounders affecting the relation among exposure, mediator and outcome is violated in observational researches, we propose a new method using the propensity score as a covariate in the high-dimensional mediation model as follows,

(3) $$\lambda_{i}\,\left(t\right)=\lambda_{0}\,\left(t\right)\,\exp\left\{\gamma^{*}\,X_{i}+\beta_{1}\,M_{1\,i}+\cdots \,\beta_{p}\,M_{p\,i}+\tilde{\varphi }\,\pi_{i}\right\},$$

(4) $$M_{k\,i}=c_{k}+\alpha_{k}\,X_{i}+e_{k\,i}+{\tilde{\phi }}_{k}\,\pi_{i},k=1,2,\cdots ,p,$$

where *π*_*i*_ is the covariate of calculated propensity score; $\tilde{\varphi }$ is the effect of the covariate on the outcome; ${\tilde{\phi }}_{k}$ is the effect of the covariate on the mediator. We will compare this with the method of adjusting all confounders as covariates and the method of ignoring confounders.

**FIGURE 2:**
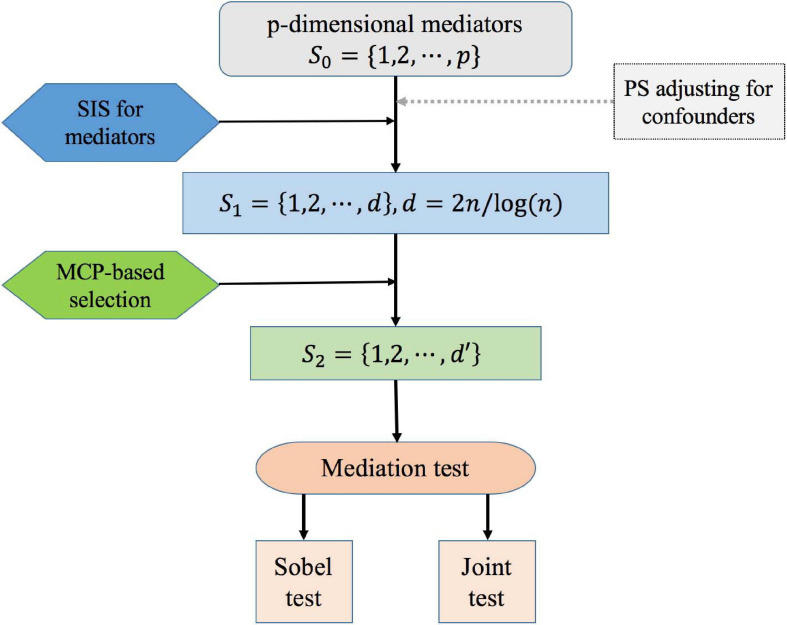
Overall workflow for high-dimensional mediation analysis. The workflow includes the main processes: (0) adjusting for confounders based on the propensity score method; (1) using SIS technique for preliminary screening; (2) conducting MCP-based variable selection; (3) testing for mediation effects. (0–3) is correspond to Step0–Step3 in the methodology.

The goal of variable selection is to identify $S=\left\{k:{\hat{\alpha }}_{k}\,{\hat{\beta }}_{k}\neq 0\right\}$, which are the significant mediators between the exposure and the outcome when the number of potential mediators *p* is much larger than the sample size *n*, and the traditional statistics methods for Cox regression analysis fail to work (Luo et al., 2020). Besides, there are confounders influence the relationship of exposure, mediators, and outcome. To solve this problem, we propose the following procedure for high-dimensional mediation analysis with confounders in survival models. The overall workflow is as follows (Figure 2):

**Step 0**: We first construct the propensity score of confounders through a logistic regression model of exposure vs. baseline confounders, and use it as a covariate in the mediation models.

**Step 1**: For *k* = 1,⋯,*p*, we select a subset *S*_1_ = {*k*:1≤*k*≤*p*} of size *d* = ⌈2*n*/*log*⁡(*n*)⌉ based on SIS method, where ⌈⋅⌉ is the ceiling function (Fan and Lv, 2008). For the mediators in *S_1* are among the top *d* strongest *P*-values for the response variable. SIS procedure has been a general technique to reduce dimensionality from high to a small scale that is below the sample size. Here we use *d* = ⌈2*n*/*log*⁡(*n*)⌉ instead of *d* = ⌈*n*/*log*⁡(*n*)⌉ to increase the probability for identifying important mediators, considering that both *α*_*k*_ and *β*_*k*_ have to be selected as nonzero to ensure a specific mediator to be selected.

**Step 2**: Among all the screened mediators *M*_*k*_, *k* ∈ *S*_1_ from Step 1, we further identify the subset $S_{2}=\left\{k:{\hat{\beta }}_{k}\neq 0\right\}$ *via* MCP-based Cox model. We obtain mediators *M_k* through the penalized log-partial likelihood optimization

$$\hat{\beta }={\text{argmax}}_{\beta }\,\left\{l_{n}\,\left(\beta \right)-\sum_{k=1}^{p}P_{\lambda }\,\left(\beta_{k}\right)\right\},k\in S_{1},$$

where $l_{n}\,\left(\beta \right)=\sum_{i=1}^{n}\delta_{i}\,\left\{P_{i}^{\text{T}}\,Q-\text{log}\,\left[\sum_{l\in R_{i}}e\,x\,p\,\left(P_{l}^{T}\,Q\right)\right]\right\}$ with the at-risk set *R*_*i*_ = {*l*:*T*_*l*_≥*T*_*i*_}, *P*_*i*_ = (*X*_*i*_,*π*_*i*_,*M*_1*i*_,⋯,[*cpsbreak*]*M*_*ki*_,⋯)^*T*^, and $Q={\left(\gamma^{*},\tilde{\varphi },\beta_{1},\cdots ,\beta_{k},\cdots \right)}^{\text{T}}$; $P_{\lambda }^{\prime }\,\left(\beta_{k}\right)=\frac{{\left(a\,\lambda -\left|\beta_{k}\right|\right)}_{+}}{a\,\lambda }$ with shape parameter *a* > 1. Breheny and Huang (2011) implemented the MCP procedure with the R package *ncvreg*.

**Step 3**: For *k* ∈ *S*_2_, a variable *M_k* is considered as a mediator between the exposure and outcome only if the indirect effect is significant. Here, we considered two methods to test the mediation effects, including the Sobel test (i.e., product method; Sobel, 1982) and the joint significant test.

Followed with the Sobel test for indirect effect, we have the *P*-value for testing the null hypothesis *H*_0_:*α*_*k*_*β*_*k*_ = 0 of no indirect effect

$$P_{r\,a\,w,k}=2\,\left\{1-\phi \,\left(\frac{\left|{\hat{\alpha }}_{k}\,{\hat{\beta }}_{k}\right|}{{\hat{\sigma }}_{\alpha_{k}\,\beta_{k}}}\right)\right\},$$

where ${\hat{\sigma }}_{\alpha_{k}\,\beta_{k}}$ is the estimate of the Sobel standard error (SE) (Sobel, 1982); ${\hat{\alpha }}_{k}$ is the ordinary least squares estimator for *α*_*k*_; ${\hat{\beta }}_{k}$ is the estimate of *β*_*k*_, by refitting regression Eq. (3) with the mediators obtained in step 2.

The joint significant test for indirect effect is based on the path-specific (i.e., *X*→*M* and *M*→*Y*) *P*-values (MacKinnon et al., 2002) and does not provide an estimate. The *P*-value for testing *H*_0_:*α*_*k*_ = 0 is given as

$$P_{r\,a\,w,\alpha_{k}}=2\,\left\{1-\phi \,\left(\frac{\left|{\hat{\alpha }}_{k}\right|}{{\hat{\sigma }}_{\alpha_{k}}}\right)\right\},$$

and the *P*-value for testing *H*_0_:*β*_*k*_ = 0 is

$$P_{r\,a\,w,\beta_{k}}=2\,\left\{1-\phi \,\left(\frac{\left|{\hat{\beta }}_{k}\right|}{{\hat{\sigma }}_{\beta_{k}}}\right)\right\}.$$

Thus, the *P*-value for the joint significance test is defined as

$$P_{r\,a\,w,k}=\text{max}\,\left(P_{r\,a\,w,\alpha_{k}},P_{r\,a\,w,\beta_{k}}\right).$$

We have the revised *P*-value *via* the Bonferroni’s method in order to adjust for multiple comparisons

$$P_{k}=\min\left\{P_{r\,a\,w,k}\cdot \left|S_{2}\right|,1\right\},$$

where |*S*_2_| is the number of elements in set *S_2*. Hence, we can reject the null hypothesis of no *IE*_*k*_ if *P*_*k*_ < 0.05, and conclude that the variable *M_k* is the significant mediator between the exposure and outcome.

**Remark 1**: Luo et al. (2020) proposed a compositional mediation framework to identify biomarkers which mediate the influence of smoking on lung cancer survival with high-dimensional candidates. They used a regression-based approach, which relies on the assumptions that there are no confounders that influence the relations between exposure and mediator, and exposure and outcome. This assumption holds if subjects are randomly assigned to levels of exposure, but generally random assignment is not possible in observational studies. We propose the use of propensity scores to adjust for confounders in high-dimensional mediation analysis in survival models.

**Remark 2**: Our method has three advantages. First, different from Luo et al. (2020), our approach is a simultaneous inference for high-dimensional mediation analysis with multiple confounders in survival models implemented with a propensity score method. The propensity score method can help remove the selection bias that may result when subjects are not randomly assigned to levels of exposure in observational studies. Second, compared with regression adjustment approach that include the confounders in mediation model directly, our method is more concise since we focus on incorporating the logit propensity score as a covariate in the mediation analysis. An advantage of propensity scores is that they reduce multiple potential confounders into a single numerical summary. Third, our method has a substantial improvement over method that does not include propensity scores.

## Simulation Studies

In this section, we evaluate the performance of the proposed mediator selection and mediation effect estimation method through simulation studies. In order to investigate how the sample size impacts the performance, three sample size levels (*N* = 300,*N* = 500,*N* = 1,000) are presented with potential mediators number *p* = 10,000. For each scenario, 500 replications of simulated data sets are conducted. Besides, we also consider two censoring rate settings of 15 and 30%.

For each subject *i*,*i* = 1,2,⋯,*N*:

1) we consider 10 confounders *Z* = (*Z*_1_,⋯,*Z*_10_)^*T*^ affecting the relationship of *X*, *M*, and *Y*, where *Z*_1_,⋯,*Z*_5_ are independently generated from the Bernoulli distribution with *Pr*(*Z*_*m*_ = 1) = 0.3,*m* = 1,2,⋯,5 and *Z*_6_,⋯,*Z*_10_ are generated from the multivariate normal distribution *N*(0,) with a covariance matrix Σ = (*σ*_*ij*_)_5×5_, *σ*_*ii*_ = 1,*i* = 1,⋯, 5 and *σ*_*ij*_ = 0.3,*i*≠*j*;
2) we generate exposure *X* as a Bernoulli distributed variable *X*_*i*_∼*Bernoulli*(*P*), where *P* = 1/[1*e*^−(*θ*^*T*^*Z*)^], and *θ* = (*θ*_1_,⋯,*θ*_10_)^*T*^ = (0.2,0.3,0.3,0.5,0.6,0.2,0.3,0.3,[*cpsbreak*]0.5,0.6)^*T*^ denote for the coefficients of confounders *Z* on *X*. The 10 confounders have varying influences on the exposure. For example, the coefficients of *Z_1* and *Z_6* are much smaller than *Z_5* and *Z*_*10*_;
3) we generate the mediator *M*_*ki*_ = *c*_*k*_ + *α*_*k*_*X*_*i*_ + *ϕ*_*k*1_*Z*_1_ + ⋯ + *ϕ*_*k*10_*Z*_10_ + *e*_*ki*_,*k* = 1,2,⋯,*p*, where *c_k* is generated from the uniform distribution *U*(0,1); (*α*_1_,⋯,*α*_8_)^*T*^ = (0.5,0.6,0.5,0.6,0.5,0.5,0,0)^*T*^ and the rest elements of α equals zero; *ϕ*_*k*_ = (*ϕ*_*k*1_,⋯,*ϕ*_*k*10_)^*T*^ = (0.3,0,0.4,0.2,[*cpsbreak*]0.5,0,0.4,0.2,0.5,0.3)^*T*^ denote the effects of *Z* on mediator *M_k*; *e_k* is generated from the standard normal distribution *N*(0,1); the correlation between mediators basically falls between 0.5 and 0.6 which is close to the real data;
4) the death time *D_i* is generated as exponential distribution with the hazard function *λ*_*i*_(*t*|*X*_*i*_,*M*_*i*_) = *λ*_0_(*t*)*exp*{*γX*_*i*_ + *β*_1_*M*_1*i*_ + ⋯ + *β*_*p*_*M*_*pi*_ + *φ*_1_*Z*_1_ + ⋯ + [*cpsbreak*]*φ*_10_*Z*_10_}, where *λ*_0_(*t*) equals 0.5; γ equals 0.5; the first eight elements of β be (*β*_1_,⋯,*β*_8_)^*T*^ = (0.6,0.6,0.5,0.5,0,0,0.5,0.5)^*T*^ and the rest elements of β equals zero; *φ* = (*φ*_1_,⋯,*φ*_10_)^*T*^ = (0,0.2,0.2,0.3,0.2,0.3,0,0.2,0.3,0.2)^*T*^ denote the effects of *Z* on *Y*;
5) the censoring time is generated through *C*_*i*_∼*U*(0,*c*_0_) with constant *c_0* chosen so that we can control the percentage of censored subjects.

To summarize, only the first four mediators have significant mediation effects, which satisfy the condition of *α*_*k*_*β*_*k*_≠0. In this part, we conduct a comparison of our proposed method with the other two approaches, including models ignoring confounders (Naïve approach) and models adjusting all 10 confounders as covariates (Z approach). We use the proposed procedure to identify significant mediators and estimate mediation effects, where the proposed approach uses the logit propensity score estimated through logistic regression as the covariate to adjust for confounding effects. Through the simulation studies, we want to demonstrate that propensity score methods can be used to adjust for confounding in the high-dimensional mediation selection and estimation.

Simulation results are presented in Tables 1–3. Table 1 evaluates the performance of mediator selection of the proposed approach in comparison to the other two approaches using the true positive rate (TPR), the number of false positive (FP), and false discovery proportion (FDP) of selection after the significance test for mediation effects based on the joint and the Sobel methods. The TPR of the proposed propensity score approach is lower than the Z approach when the sample size is 300, but performs similarly to the Z approach as the sample size increases. And the proposed method has lower FP and FDP rates than the Z approach. The Naïve approach has lower TPR and higher FP and FDP rates, indicating the deficiency in identifying significant mediators due to confounding effects. Take sample size 500 as an example, the FP and FDP rates based on the joint test are 0.004 and 0.0008 for the proposed approach; 0.026 and 0.0056 for the Z approach; and 4.342 and 0.5168 for the Naïve approach. Selection results based on the joint test are similar. Besides, as the censoring rate increases, the TPR rates decrease, especially for the lower sample size. Similar results can be seen for the setting with a 30% censoring rate.

**TABLE 1:** Accuracy of mediator selection (*p* = 10,000, with 500 replications).

**Cen = 15%**	**Methods**	**Sobel test**			**Joint test**		
		**TPR**	**FP**	**FDP**	**TPR**	**FP**	**FDP**
*N* = 300	PS	0.6025	0	0	0.6890	0.0100	0.0025
	Naïve	0.6580	4.4780	0.4982	0.6580	5.0860	0.5721
	Z	0.6670	0	0	0.7800	0.0240	0.0060
*N* = 500	PS	0.9610	0.0020	0.0004	0.9690	0.0040	0.0008
	Naïve	0.9425	3.6400	0.4745	0.9425	4.3420	0.5168
	Z	0.9565	0.0020	0.0004	0.9695	0.0260	0.0056
*N* = 1,000	PS	1	0.0100	0.0020	1	0.0100	0.0020
	Naïve	0.9995	3.3420	0.4401	0.9995	3.6460	0.4593
	Z	1	0.0100	0.0020	1	0.0280	0.0056

**Cen = 30%**	**Methods**	**TPR**	**FP**	**FDP**	**TPR**	**FP**	**FDP**

*N* = 300	PS	0.5370	0.0020	0.0005	0.6565	0.0080	0.0021
	Naïve	0.6370	3.6060	0.5469	0.6370	5.3460	0.6474
	Z	0.5825	0	0	0.7450	0.0220	0.0058
*N* = 500	PS	0.9505	0.0020	0.0004	0.9650	0.0080	0.0017
	Naïve	0.9235	3.5900	0.4778	0.9235	4.3940	0.5285
	Z	0.9460	0	0	0.9695	0.0340	0.0069
*N* = 1,000	PS	1	0.0080	0.0016	1	0.0100	0.0020
	Naïve	0.9995	3.6160	0.4581	0.9995	3.9020	0.4756
	Z	1	0.0040	0.0008	1	0.0260	0.0052

*PS, method of using the propensity score as the covariate; Naïve, method of ignoring confounders; Z approach, method of using confounders as covariates. TPR, true positive rates; FP, false positive; FDP, false discovery proportion (=V/R, where V is the number of false discoveries and R is the number of total discoveries); all the three measures are the average value over 500 times.*

Tables 2, 3 show the estimation of mediation effects with censoring rate by 15 and 30%, respectively. The bias of the indirect effect estimator using the PS approach is very small. The Naïve approach is biased severely. It is important to note that the proposed method even has slightly better performance than the Z approach including all confounders as covariates in the estimation of indirect effects.

**TABLE 2:** Estimation of log hazard mediation effects: *α*_*k*_*β*_*k*_ (Cen = 15%).

**Cen = 15%**	***N* = 300**			***N* = 500**			***N* = 1,000**		
(*α*_*k*_,*β*_*k*_)	**PS**	**Naïve**	**Z**	**PS**	**Naïve**	**Z**	**PS**	**Naïve**	**Z**
(0.5, 0.6) = 0.30 (MSE)	0.2895 (0.0088)	0.7712 (0.2484)	0.2925 (0.0100)	0.2960 (0.0049)	0.8473 (0.3156)	0.3117 (0.0062)	0.3077 (0.0026)	0.8545 (0.3145)	0.3151 (0.0026)
(0.6, 0.6) = 0.36 (MSE)	0.3501 (0.0096)	0.8333 (0.2547)	0.3574 (0.0127)	0.3559 (0.0058)	0.8932 (0.3024)	0.3765 (0.0075)	0.3682 (0.0030)	0.9111 (0.3122)	0.3728 (0.0031)
(0.5, 0.5) = 0.25 (MSE)	0.2426 (0.0061)	0.6614 (0.1901)	0.2680 (0.0082)	0.2499 (0.0037)	0.7032 (0.2192)	0.2626 (0.0048)	0.2576 (0.0019)	0.7075 (0.2161)	0.2592 (0.0018)
(0.6, 0.5) = 0.30 (MSE)	0.2907 (0.0073)	0.7034 (0.1885)	0.3146 (0.0102)	0.3045 (0.0044)	0.7513 (0.2191)	0.3228 (0.0060)	0.3120 (0.0021)	0.7564 (0.2149)	0.3130 (0.0021)
(0.5, 0) = 0 (MSE)	– (–)	0.3002 (0.0902)	– (–)	– (–)	– (–)	– (–)	– (–)	0.1246 (0.0155)	– (–)
(0.5, 0) = 0 (MSE)	– (–)	– (–)	– (–)	0.0724 (0.0052)	0.1789 (0.0320)	– (–)	– (–)	0.1588 (0.0253)	0.0688 (0.0047)
(0, 0.5) = 0 (MSE)	0.0037 (0.0048)	0.4406 (0.2075)	0.0201 (0.0061)	0.0040 (0.0026)	0.4727 (0.2305)	0.0079 (0.0028)	0.0031 (0.0015)	0.4769 (0.2309)	0.0031 (0.0014)
(0, 0.5) = 0 (MSE)	0.0026 (0.0051)	0.4479 (0.2143)	0.0102 (0.0059)	0.0022 (0.0029)	0.4711 (0.2289)	0.0006 (0.0032)	0.0017 (0.0016)	0.4746 (0.2293)	0.0020 (0.0016)
(0, 0) = 0 (MSE)	– (–)	– (–)	– (–)	– (–)	0.1624 (0.0263)	0.0247 (0.0006)	– (–)	0.0882 (0.0078)	– (–)
(0, 0) = 0 (MSE)	– (–)	– (–)	– (–)	– (–)	– (–)	0.0135 (0.0002)	– (–)	– (–)	– (–)

*PS, method of using the propensity score as the covariate; Naïve, method of ignoring confounders; Z approach, method of using confounders as covariates. MSE, mean square error; –, means the not available value.*

**TABLE 3:** Estimation of log hazard mediation effects: *α*_*k*_*β*_*k*_ (Cen = 30%).

**Cen = 30%**	***N* = 300**			***N* = 500**			***N* = 1,000**		
(*α*_*k*_,*β*_*k*_)	**PS**	**Naïve**	**Z**	**PS**	**Naïve**	**Z**	**PS**	**Naïve**	**Z**
(0.5, 0.6) = 0.30 (MSE)	0.2793 (0.0096)	0.7589 (0.2415)	0.2982 (0.0108)	0.2932 (0.0053)	0.8362 (0.3085)	0.3139 (0.0070)	0.3081 (0.0028)	0.8597 (0.3214)	0.3197 (0.0034)
(0.6, 0.6) = 0.36 (MSE)	0.3431 (0.0113)	0.8289 (0.2546)	0.3666 (0.0146)	0.3528 (0.0059)	0.8851 (0.2978)	0.3819 (0.0084)	0.3689 (0.0032)	0.9179 (0.3213)	0.3839 (0.0040)
(0.5, 0.5) = 0.25 (MSE)	0.2377 (0.0069)	0.6745 (0.2054)	0.2697 (0.0098)	0.2480 (0.0040)	0.6983 (0.2176)	0.2649 (0.0057)	0.2571 (0.0019)	0.7099 (0.2199)	0.2618 (0.0022)
(0.6, 0.5) = 0.30 (MSE)	0.2803 (0.0084)	0.7082 (0.2021)	0.3241 (0.0130)	0.3018 (0.0046)	0.7449 (0.2162)	0.3226 (0.0067)	0.3121 (0.0023)	0.7626 (0.2225)	0.3184 (0.0029)
(0.5, 0) = 0 (MSE)	– (–)	– (–)	– (–)	– (–)	– (–)	0.0818 (0.0067)	– (–)	– (–)	0.0390 (0.0015)
(0.5, 0) = 0 (MSE)	– (–)	– (–)	– (–)	– (–)	– (–)	0.0925 (0.0085)	– (–)	0.1301 (0.0169)	0.0730 (0.0053)
(0, 0.5) = 0 (MSE)	0.0056 (0.0046)	0.4388 (0.2087)	0.0080 (0.0061)	0.0043 (0.0026)	0.4657 (0.2259)	0.0043 (0.0029)	0.0034 (0.0015)	0.4798 (0.2346)	0.0034 (0.0015)
(0, 0.5) = 0 (MSE)	0.0016 (0.0051)	0.4394 (0.2084)	0.0031 (0.0061)	0.0015 (0.0029)	0.4631 (0.2232)	0.0021 (0.0033)	0.0018 (0.0016)	0.4743 (0.2297)	0.0021 (0.0016)
(0, 0) = 0 (MSE)	– (–)	– (–)	0.0391 (0.0015)	– (–)	– (–)	– (–)	– (–)	0.0977 (0.0095)	0.0062 (0.0001)
(0, 0) = 0 (MSE)	– (–)	– (–)	0.0147 (0.0002)	– (–)	– (–)	– (–)	– (–)	– (–)	0.0008 (0.0000)

*PS, method of using the propensity score as the covariate; Naïve, method of ignoring confounders; Z approach, method of using confounders as covariates. MSE, mean square error; –, means the not available value.*

In summary, the results demonstrate that the bias of the mediation effect estimator of our proposed methods for high-dimensional mediation analysis using the calculated propensity score to adjust confounding influence is nearly unbiased. Besides, with the increase of sample size, the ability in mediator selection including TPR, the number of FP, and FDP shows good performance as well as the Z approach. The Naïve approach ignoring the confounders produces a severe bias in both mediator selection and mediation effects estimation. Compared with the classical regression method for mediation analysis with confounders, the procedure we proposed is more concise and efficient.

## Real Data Analysis

As we know, smoking is an important risk factor for lung cancer, one of the deadliest cancer worldwide (Herbst et al., 2008). With the development of sequencing technology, both Illumina Infinium HumanMethylation27 and HumanMethylation450 are widely used platforms that allow measuring high-dimensional DNA methylation levels of roughly 27 and 450 k respectively(Bibikova et al., 2011). As the individual level phenotype and genotype data are available, researchers have indicated that methylation markers are acting as mediators between smoking and lung function or lung cancer patient’s overall survival (Zhang et al., 2016; Luo et al., 2020). The TCGA (The Cancer Genome Atlas) lung cancer cohort study had been used for mediation analysis to identify the methylation markers (Luo et al., 2020). However, the assumption that samples are randomly assigned to the smoking or non-smoking group is violated. Hence, it is of great importance to adjust for confounding effects when conducting high-dimensional mediation analysis.

We apply the proposed method using the calculated propensity score as a covariate in high-dimensional mediation analysis with survival outcome to a lung cancer dataset including lung squamous cell carcinoma and lung adenocarcinoma. There are 1,299 lung cancer patients aged 33–90 years and 907 of them had DNA methylation profile measured using the Illumina Infinium HumanMethylation 450 platform. DNA methylation values were recorded for each array probe in each sample *via* BeadStudio software. A total of 365,307 probes were included in the analysis.

To identify the potential methylation mediators between the tobacco smoking and the overall survival, we apply the high-dimensional mediator model with smoking status assessed at their initial diagnosis (smoker/non-smoker) as the exposure variable, DNA methylation measured concurrently as the high-dimensional mediators, and the survival time as the outcome variable. The overall survival time is defined as the number of days from the initial diagnosis to the death or the last follow-up date. Subjects with no observed time, exposure, and other covariates are excluded; there are 696 patients with 269 deaths left. Covariates including age at initial diagnosis, gender, and radiotherapy (yes/no) are considered.

We first adjust for the baseline confounders including age, gender, and radiotherapy using the calculated propensity score. Due to the fact that the relationships between methylation and the outcome are much stronger than those between exposure and methylation in the analysis data set, we add top *d* = 2*n*/log(*n*) CpGs using SIS method based on the path from smoking to the methylation in order to improve the probability to recognize significant mediators. Then, we run a variable selection on the CpGs screened in the above step. Finally, we carry out the significance test for the mediation effects.

The analysis results are presented in Table 4. We identify CpGs mediating the relationship between smoking and the overall survival of lung cancer patients with Bonferroni’s adjusted *P* < 0.05. Since smoking generally increases the risk of progression to death and reduces the overall survival of lung cancer patients with the total effect of 1.3436 (95% CI: 1.0377–1.7400), we focus on the mediators with the log-hazard indirect effect *αβ* = 0 (smoking increases the mortality). Our method finds two CpGs (cg21926276 and cg20707991) mediating the relationship of smoking and risk of progression to death, while methods including all confounders as covariates and methods ignoring confounders only find cg20707991 to be a significant mediator. The methylation site cg21926276 has been reported as a mediator of smoking and the risk of progression to death (Luo et al., 2020). All the two genes in which methylation sites locate are associated with lung cancer or tumor growth in previous studies. For example, the gene H19 (cg21926276 locate) is related to both lung cancer and tumor growth, methylation of which has been thought of as a sensitive marker of tobacco history (Bouwland-Both et al., 2015; Matouk et al., 2015). The gene PTPRN2 (cg20707991 locate) is also associated with lung cancer and survival of cancer patients (Anglim et al., 2008; Wielscher et al., 2015). Besides confirming the previously reported genes, cg20707991 is identified as a novel marker for the survival of lung cancer patients.

**TABLE 4:** Summary of selected CpGs with estimators ($\hat{\alpha }\,\hat{\beta }>0$) and *P*-values for significant mediators.

**Methods**	**CpGs**	**Chromosome**	**Gene**	$\hat{\alpha }$	$\hat{\beta }$	***P*(Sobel)**	***P*(Joint)**
Proposed	cg21926276	chr11	H19	–0.06	–3.21	6.69e–03	1.75e-04
	cg20707991	chr7	PTPRN2	–0.06	–2.12	5.36e–02	1.28e–02
Z	cg20707991	chr7	PTPRN2	–0.06	–2.40	2.49e–03	4.82e–05
Naïve	cg20707991	chr7	PTPRN2	–0.06	–1.01	2.58e–02	1.61e–02

The CpGs are the DNA methylation sites. Chromosomes and Genes are where the CpGs locate. $\hat{\alpha }$ is the estimation of the effect of exposure on methylation. $\hat{\beta }$ is the estimation of the effect of methylation on the risk of progression to death. *P*(Sobel) is the Sobel test *P*-values and *P*(Joint) is the joint test *P*-values, which are corrected by Bonferroni’s method.

Based on the above analysis, compared with non-smokers, the risk of death for those smokers is 1.3436 (95% CI: 1.0377–1.7400). Mediation analysis using Cox proportional hazards model discovers that the effect of having serious smoking history on the increased risk of progression to death is mediated through methylation markers including cg21926276 and cg20707991; the hazard ratio for each mediator is 1.2093 (95% CI: 1.2019–1.2167) and 1.1388 (95% CI: 1.1339–1.1438), respectively. Interventions can be explored on these markers to improve medical care for the detection and treatment of lung cancer among smokers.

To sum up, through the mediation analysis of smoking, DNA methylation, and the survival time of the lung cancer patients, we found two CpGs mediating the smoking and the mortality. Our findings not only were in line with previous studies which found that the gene that CpGs locate were important biomarkers for lung cancer, but also uncovered the mediation role of the markers connecting the smoking exposure and the survival time.

## Discussion

The motivation of this study is that the assumption of no confounders affecting the relationship of exposure, mediators and outcome in the classical mediation model is difficult to be satisfied with observational studies. Hence, how to adjust these confounders is an important and practical question. The propensity score method can summarize a large scale of confounders into a single value which is more concise than the methods with a regression adjustment for all the potential confounders. Thus, motivated by the above facts, we develop a new method that using the propensity score as a covariate to adjust for confounding effects in high-dimensional mediation models.

In this article, we focus on how to adjust for confounding influences when the exposure is not randomly assigned in observational studies. We propose a new model for high-dimensional mediation analysis using propensity score methods to adjust for confounding effects. To identify the significant mediators from high-dimensional potential candidate variables, we mainly combine the sure screening technique, MCP-based penalty, and Sobel and joint methods for significance tests. We evaluate the performance of the proposed procedure *via* several simulation studies and a real data application.

Compared with the mediation analysis which includes all the confounders as covariates, our proposed approach for high-dimensional mediation analysis using the calculated propensity score to adjust confounding influence would be an improvement in mediator selection and indirect effect estimation. The simulation results also show that the proposed method can obtain a nearly unbiased estimation for indirect effects. It is also interesting to note that if confounders are omitted from the model, then the estimates for mediation effects will be severely biased. In conclusion, we suggest using the calculated propensity score to adjust for confounders among the exposure, mediators, and the outcome when evaluating mediation.

As mentioned previously, propensity score methods have many other applications, such as matching, weighting, and sub-classification. It is of interest to explore the performance of high-dimensional mediation selection and estimators using propensity score weighting. Also, the propensity score in our current approach is only valid for single exposure. Analysis approach for the high-dimensional mediators with more than two exposure status is still to be developed. The present simulation results do not address the cases that confounders only affect mediators and the outcome. It is of future interest to developed methods involving estimating propensity score for high-dimensional mediators. Of note, the Sobel test and the joint significant test we used are conservative, which paves the way for developing a more powerful test method, such as the Divide-Aggregate Composite null Test (DACT; Liu et al., 2021). The DACT method is especially useful for the composite null hypothesis of no mediation effect in large-scale genome-wide epigenetic studies. It is desirable to consider such a powerful test method for mediation effects in the future research.

## Data Availability Statement

The TCGA (The Cancer Genome Atlas) lung cancer data we used in our real data analysis can be found in (https://xenabrowser.net/) without limitation. Our procedure is implemented using the R tool. The corresponding R code can be found at https://github.com/luo-chengwen/HIMAsurvival-PS.

## Author Contributions

CL and ZY implemented the method, drafted the manuscript, conceived the idea, designed the study, and implemented the code. YC, TW, and YM were involved in the data analysis. All authors read and approved the final manuscript.

## Conflict of Interest

The authors declare that the research was conducted in the absence of any commercial or financial relationships that could be construed as a potential conflict of interest.
